# Effectiveness of Behavioural Modification Techniques in Children Having Intellectual Disability Pre and Post Evaluation

**DOI:** 10.1192/bjo.2025.10229

**Published:** 2025-06-20

**Authors:** Irum Siddique

**Affiliations:** East London Foundation trust, Bedford, United Kingdom

## Abstract

**Aims:** To find out the effectiveness of different strategies of behaviour modification techniques on children having intellectual disability in specific time period under controlled setting and compare pre and post scores.

**Methods:** 36 children diagnosed with intellectual disability were included in current studies, comprised of 20 boys and 16 girls. A diagnostic criterion of DSM–V for intellectual disability was applied to diagnose the children. Portage Guide to Early Education (PGEE) was selected to then find out the developmental age of children and in some of the cases to find out the functional level of intellectually impaired children. For diagnostic point of view intelligence test including Slosson Intelligence test was administered for screening children and Coloured Progressive Matrices was used to find out the level of mental maturity. Behaviour modification techniques that were selected for administration of IEPs in specific areas were reinforcement techniques, shaping, chaining, prompt-fading and negative punishment. The training programme consists of five days per week and four hours per day by the help of team of trained professionals; specifically all were trained clinical psychologists.

**Results:** Repeated measure t-test has shown significant statistical difference between pre and post ratings of the Intellectually Disabled children on all the domains including Self Help, motor, cognitive, language and socialization of PGEE, results shows the difference respectively; t (35)=−8.82, −8.393, −7.496. −7.541 and −7.4295, p=0.000 two tailed on above mentioned domains. Intellectually disabled children scored higher in post ratings (M=52.25, SD=15.39); (M=54.58, SD=12.46); (M=37.25, SD=18.13); (M=43.44, SD=19.00); (M=52.56, SD=17.33) on Self Help, motor, cognitive. Language and socialization domains of PGEE then pre test rating (M=44.67, SD=16.44); (M=44.78, SD=13.52); (M=29.53, SD=18.13); (M=36.47, SD=19.74); (M=43.75, SD=16.83).

**Conclusion:** IEPs do work effectively in dealing with children having special needs with use of behavioural intervention as it was found in our research with drastic change in post rating of each adaptive area like self-help, motor, cognitive, language and socialization.

