# Effectiveness of Behavioural Modification Techniques in children Having Intellectual Disability Pre and Post Evaluation – CORRIGENDUM

**DOI:** 10.1192/bjo.2025.10890

**Published:** 2025-10-09

**Authors:** Samreen Afzal, Irum Siddique, Imtiaz Ahmed Dogar, Waqar Azeem

In the original publication of this abstract, several of the co-authors were missing from the author list. The correct author list is as follows:

Samreen Afzal, Irum Siddique, Dr Imtiaz Ahmed Dogar and Dr Waqar Azeem

The abstract has been updated to amend this, and this corrigendum published.
